# Supplementary material for: Gut bacteria induce oviposition preference through ovipositor recognition in fruit fly
Source: Commun Biol. 2022 Sep 15;5:973. doi: 10.1038/s42003-022-03947-z (PMC9477868; doi:10.1038/s42003-022-03947-z)
Supplement: Supplementary file 2 — Supplementary Information [file 42003_2022_3947_MOESM2_ESM.pdf]

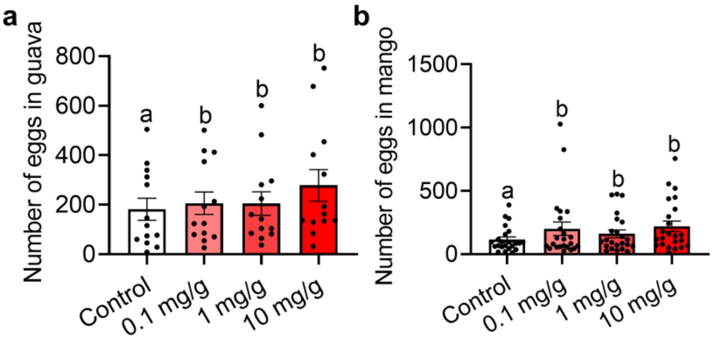

Supplementary Figure 1 Comparison of number of eggs laid in purees and purees mixed with3-HA. (a) Eggs laid in guava purees and purees mixed with 3-HA (n = 13 replicates, Kendall W = 0.28, χ^2 = 10.938, P = 0.012). (b) Eggs laid in mango purees and purees mixed with 3-HA (n = 23 replicates, Kendall W = 0.136, χ^2 = 9.393, P = 0.024). Number of blue dots in each bar indicated the replicates. Different letters above the error bars indicate significant differences at the 0.05 level with the Kendall nonparametric test. Data in bar plots show mean values ±SEM.

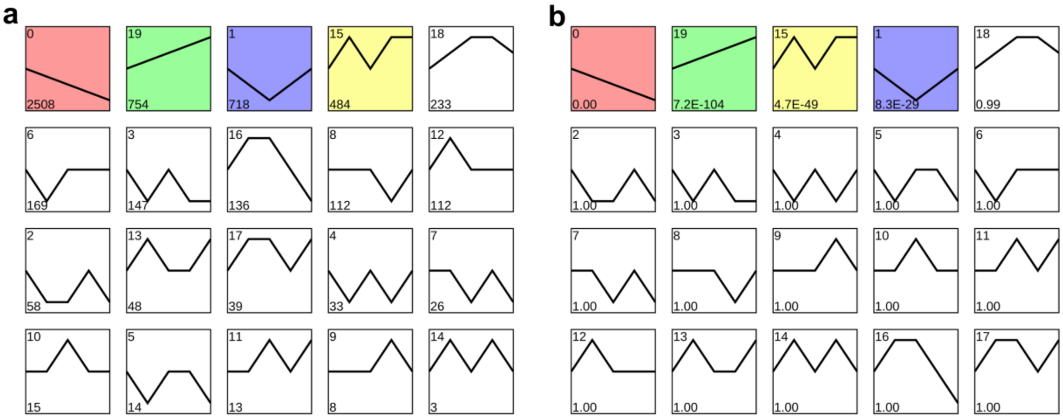

Supplementary Figure 2 Number of genes in different expression patterns (a) and statistical analysis (b).

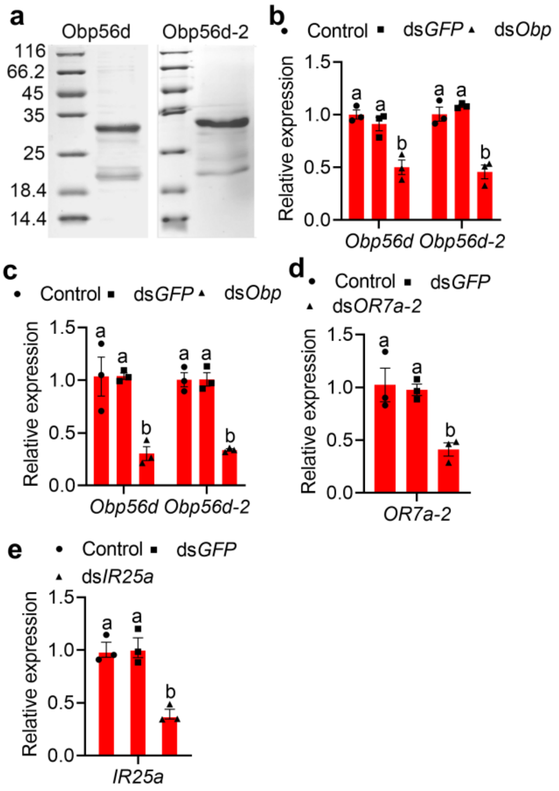

Supplementary Figure 3 Gene expressions analysis. (a) Recombinant protein of Obp56d and Obp56d-2 verification. (b) Expression of *Obp56d* and *Obp56d-2* gene 24 h after their dsRNA was injected individually (*Obp56d*: F = 20.07, df = 2, 6, P = 0.0022; *Obp56d-2*: F = 39.16, df = 2, 6, P = 0.0004). (c) Expression of *Obp56d* and *Obp56d-2* gene 24 h after their dsRNA was injected together (*Obp56d*: F = 13.61, df = 2, 6, P = 0.0059; *Obp56d-2*: F = 51.5, df = 2, 6, P = 0.0002). (d) Expression of *OR7a-2* gene 24 h after its dsRNA was injected (F = 10.78, df = 2, 6, P = 0.0103). (e) Expression of *IR25a* gene 24 h after its dsRNA was injected (F = 23.83, df = 2, 6, P = 0.0014). Number of black dots, squares and triangles in each bar indicated the replicates (n = 3 replicates). Different letters above the error bars indicate significant differences at the 0.05 level analyzed by ANOVA followed by Tukey’s test. Data in bar plots show mean values ± SEM. Detail information for sample sizes can be found in Supplementary Data 2.

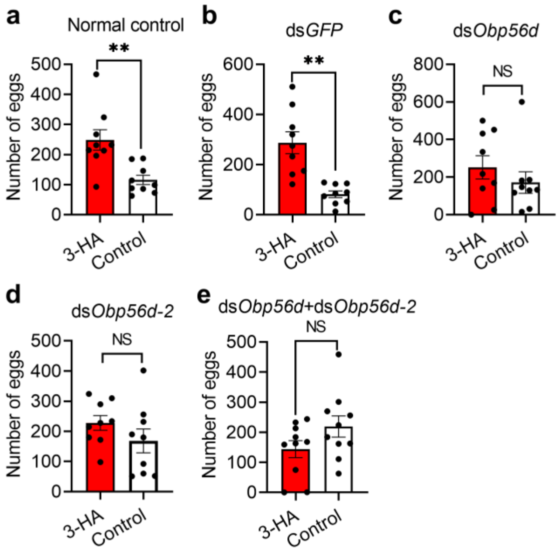

Supplementary Figure 4 Comparison of egg number in guava puree and guava puree added 3-HA. (a) Comparison of the number of eggs laid by normal flies in guava puree and guava puree added 3-HA (n = 9 replicates, P = 0.0039, Sum of signed ranks (W) = -45). (b) Comparison of the number of eggs laid by ds*GFP* injected flies in guava puree and guava puree added 3-HA (n = 9 replicates, P = 0.0039, Sum of signed ranks (W) = -45). (c) Comparison of the number of eggs laid by ds*Obp56d* injected flies in guava puree and guava puree added 3-HA (n = 9 replicates, P = 0.375, Sum of signed ranks (W) = -16). (d) Comparison of the number of eggs laid by ds*Obp56d-2* injected flies in guava puree and guava puree added 3-HA (n = 9 replicates, P = 0.25, Sum of signed ranks (W) = -21). (e) Comparison of the number of eggs laid by ds*Obp56d* and ds*Obp56d-2* injected flies in guava puree and guava puree added 3-HA (n = 10 replicates, P = 0.4922, Sum of signed ranks (W) = 15). The data was analyzed with Wilcoxon matched-pairs signed rank test. "NS" no significance, ** $P < 0.01$. Data in bar plots show mean values ± SEM.

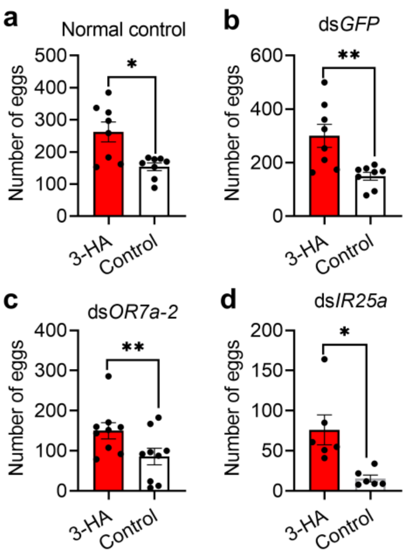

Supplementary Figure 5 Comparison of egg number in guava puree and guava puree added 3-HA. (a) Comparison of the number of eggs laid by normal flies in

guava puree and guava puree added 3-HA (n = 8 replicates, P = 0.0156, Sum of signed ranks (W) = -34). (b) Comparison of the number of eggs laid by ds*GFP* injected flies in guava puree and guava puree added 3-HA (n = 8 replicates, P = 0.0078, Sum of signed ranks (W) = -36). (c) Comparison of the number of eggs laid by ds*OR7a-2* injected flies in guava puree and guava puree added 3-HA (n = 9 replicates, P = 0.0078, Sum of signed ranks (W) = -36). (d) Comparison of the number of eggs laid by ds*IR25a* injected flies in guava puree and guava puree added 3-HA (n = 6 replicates, P = 0.0313, Sum of signed ranks (W) = -21). The data was analyzed with Wilcoxon matched-pairs signed rank test. * $P < 0.05$, ** $P < 0.01$. Data in bar plots show mean values ±SEM.

Supplementary Table 1 Primers used in qRT-PCR experimental

Primer name	Sequence (5'-3')	Length (bp)
α-TUB-F	CGCATTCATGGTTGATAACG	184
α-TUB-R	GGGCACCAAGTTAGTCTGGA	
ACT5-F	CAACTCACCCGCAATGTATG	237
ACT5-R	CGCTCAGCAGTGGTTGTAAA	
Obp19d-F	TAGTGGTTTTACCTGCGCGT	92
Obp19d-R	AGCAAAGCTGCAGATAGCCA	
Obp56d-2-F	TTCCGAAGAGGCGATTCGTG	88
Obp56d-2-R	CTCTTGCATGCACTTGGAGA	
Obp56d-F	TTCGCTGTTGCTGTTTTGCT	99
Obp56d-R	TGCAGCGTGTACCTTTTGTT	
OR7a-2-F	CTTTCTGTGCTACTCGGCCA	112
OR7a-2-R	TATAAACTCCGTGCGGGAGC	
IR25a-F	ATGCAGGAAGCCCAGTTACC	71
IR25a-R	GTGGCTGAAGTTGAGTTGCG	

Supplementary Table 2 Primers used for recombinant proteins and dsRNA synthesis

	**Sequence (5'-3')**
Re-*Obp56d*-F	GGTGGTGGATCCGAATTCCGGACTATGCAGGAAGGCGTTGGCAAACTGACCGAAGAACA
Re-*Obp56d*-R	GGTGGTGGTGCTCGAGTGCGGCCTTATTAAAACACAAAGCTTTCATGGCCTGCTGCATA
Re-*Obp56d*-2 F	GGTGGTGGATCCGAATTCCGGACTATGCAGGAAGGCGTTAATAAACTGACCGAAGAACA
Re-*Obp56d*-2 R	GGTGGTGGTGCTCGAGTGCGGCCTTATTAAAAATCCAGGCCTTCATGACCGGCGGCAT
ds*Obp19d*-F	TAATACGACTCACTATAGTTCGCTGTTGCTGTTTTGCTA
ds*Obp19d*-R	TAATACGACTCACTATAGTAAGGCCTTTGGACTTCGCA
ds*Obp56d*-2 F	TAATACGACTCACTATAGTTCGCTGTTGCTGTTTTGCTA
ds*Obp56d*-2 R	TAATACGACTCACTATAGATCCAAGCCCTCGTGCC
ds*Obp56d*-F	TAATACGACTCACTATAGTTCGCTGTTGCTGTTTTGCTA
ds*Obp56d*-R	TAATACGACTCACTATAGAAAACAAAGCTCTCATGCCCA
ds*OR7a-2*-F	TAATACGACTCACTATAGCTACCACTTGCCGATTGATGC
ds*OR7a-2*-R	TAATACGACTCACTATAGCGAAGGCATGACTAAGCCCA
ds*IR25a*-F	TAATACGACTCACTATAGAGCCCCTACAGCTACCAGAA
ds*IR25a*-R	TAATACGACTCACTATAGCACCGGTGTATCGAGACGAG

Note: The blue sequence is the homologous sequence of vector ; The red sequence is T7 promoter.
