# Supplementary material for: Gut bacteria induce oviposition preference through ovipositor recognition in fruit fly
Source: Commun Biol. 2022 Sep 15;5:973. doi: 10.1038/s42003-022-03947-z (PMC9477868; doi:10.1038/s42003-022-03947-z)
Supplement: Supplementary file 3 — Description of Additional Supplementary Files [file 42003_2022_3947_MOESM3_ESM.pdf]

Description of Additional Supplementary Files

File name: Supplementary Data 1
Description: : Differentially expressed genes exhibited an upward trend in ovipositors from eclosion to sexual maturity.

File name: Supplementary Data 2
Description: The source data behind the graphs in the paper.

File name: Supplementary Movie 1
Description: Egg laying behavior observation.
