# Supplementary material for: Gut bacteria induce oviposition preference through ovipositor recognition in fruit fly
Source: Commun Biol. 2022 Sep 15;5:973. doi: 10.1038/s42003-022-03947-z (PMC9477868; doi:10.1038/s42003-022-03947-z)
Supplement: Supplementary file 7 — Reporting Summary [file 42003_2022_3947_MOESM7_ESM.pdf]

nature portfolio

Corresponding author(s): Daifeng Cheng

Last updated by author(s): Aug 25, 2022

Reporting Summary

Nature Portfolio wishes to improve the reproducibility of the work that we publish. This form provides structure for consistency and transparency in reporting. For further information on Nature Portfolio policies, see our Editorial Policies and the Editorial Policy Checklist.

Statistics

For all statistical analyses, confirm that the following items are present in the figure legend, table legend, main text, or Methods section.

n/a	Confirmed	
☐	☒	The exact sample size (*n*) for each experimental group/condition, given as a discrete number and unit of measurement
☒	☐	A statement on whether measurements were taken from distinct samples or whether the same sample was measured repeatedly
☒	☐	The statistical test(s) used AND whether they are one- or two-sided<br>*Only common tests should be described solely by name; describe more complex techniques in the Methods section.*
☐	☒	A description of all covariates tested
☐	☒	A description of any assumptions or corrections, such as tests of normality and adjustment for multiple comparisons
☐	☒	A full description of the statistical parameters including central tendency (e.g. means) or other basic estimates (e.g. regression coefficient) AND variation (e.g. standard deviation) or associated estimates of uncertainty (e.g. confidence intervals)
☐	☒	For null hypothesis testing, the test statistic (e.g. *F*, *t*, *r*) with confidence intervals, effect sizes, degrees of freedom and *P* value noted<br>*Give P values as exact values whenever suitable.*
☒	☐	For Bayesian analysis, information on the choice of priors and Markov chain Monte Carlo settings
☒	☐	For hierarchical and complex designs, identification of the appropriate level for tests and full reporting of outcomes
☒	☐	Estimates of effect sizes (e.g. Cohen's *d*, Pearson's *r*), indicating how they were calculated

Our web collection on statistics for biologists contains articles on many of the points above.

Software and code

Policy information about availability of computer code

Data collection	An index of the reference genome of B. dorsalis was built, and paired-end clean reads were mapped to the reference genome using HISAT2. 2.4 with “-rna-strandness RF” and other parameters set as a default. To evaluate transcript expression abundances, StringTie software was applied for calculation of the normalized gene expression value FPKM. Then, gene differential expression analysis was performed with DESeq2 software. To examine the expression pattern of the differentially expressed genes, the expression data of each sample were normalized and then clustered with Short Time-series Expression Miner software (STEM). GC/MSD ChemStation (Agilent7890B-5977B), GC-EAD (Agilent, Syntech)
Data analysis	SPSS 19.0, Graphpad prism 8.0

For manuscripts utilizing custom algorithms or software that are central to the research but not yet described in published literature, software must be made available to editors and reviewers. We strongly encourage code deposition in a community repository (e.g. GitHub). See the Nature Portfolio guidelines for submitting code & software for further information.

Data

Policy information about availability of data

All manuscripts must include a data availability statement. This statement should provide the following information, where applicable:

- Accession codes, unique identifiers, or web links for publicly available datasets
- A description of any restrictions on data availability
- For clinical datasets or third party data, please ensure that the statement adheres to our policy

All data needed to evaluate the conclusions in the paper are present in the paper and/or the Supplementary Materials. Raw data, replicates and statistical analysis methods in the figures are listed in Supplementary Data 2. Supplementary Movie 1 was uploaded in Figshare (https://figshare.com/articles/media/Gut_bacteria_induced_oviposition_preference_through_ovipositor_recognition/20575248). The transcriptome data of ovipositor have been uploaded to National Genomics Data Center with accession number being PRJCA004790. Accession number of Obp56d, Obp56d-2, Obp19d, OR7a-2 and IR25a in GenBank were XM_011198997.3, XM_011198998.2, XM_011213044.3, KP743713.1 and XM_011209493.3, respectively.

Human research participants

Policy information about studies involving human research participants and Sex and Gender in Research.

Reporting on sex and gender	NA
Population characteristics	NA
Recruitment	NA
Ethics oversight	NA

Note that full information on the approval of the study protocol must also be provided in the manuscript.

Field-specific reporting

Please select the one below that is the best fit for your research. If you are not sure, read the appropriate sections before making your selection.

☒ Life sciences ☐ Behavioural & social sciences ☐ Ecological, evolutionary & environmental sciences

For a reference copy of the document with all sections, see nature.com/documents/nr-reporting-summary-flat.pdf

Life sciences study design

All studies must disclose on these points even when the disclosure is negative.

Sample size	In all experiments, each group has at least three biological replicates.
Data exclusions	No data was excluded in analysis.
Replication	All attempts at replication were successful.
Randomization	All flies used in the study were reared at the same condition and selected randomly for experiment.
Blinding	Blinding is not relevant to the study. Because the organism used is fruit fly.

Reporting for specific materials, systems and methods

We require information from authors about some types of materials, experimental systems and methods used in many studies. Here, indicate whether each material, system or method listed is relevant to your study. If you are not sure if a list item applies to your research, read the appropriate section before selecting a response.

Materials & experimental systems

n/a	Involved in the study
☒	☐ Antibodies
☒	☐ Eukaryotic cell lines
☒	☐ Palaeontology and archaeology
☐	☒ Animals and other organisms
☒	☐ Clinical data
☒	☐ Dual use research of concern

Methods

n/a	Involved in the study
☒	☐ ChIP-seq
☒	☐ Flow cytometry
☒	☐ MRI-based neuroimaging

Animals and other research organisms

Policy information about studies involving animals; ARRIVE guidelines recommended for reporting animal research, and Sex and Gender in Research

Laboratory animals	Not applicable.
Wild animals	Not applicable.
Reporting on sex	Not applicable.
Field-collected samples	Not applicable.
Ethics oversight	Not applicable.

Note that full information on the approval of the study protocol must also be provided in the manuscript.
